# Anatomical Variations and Surgical Implications of the Cervical Branch of the Facial Nerve: A Systematic Review

**DOI:** 10.1093/asjof/ojag079

**Published:** 2026-06-29

**Authors:** Ahmad Bogari, Lenah AlFadhil, Khalid Saad Alqarni, Naif Fnais, Mohammed Jomah

## Abstract

The cervical branch of the facial nerve (CBFN) innervates the platysma and contributes to lower facial animation. Due to its anatomical variability and proximity to critical structures, including the marginal mandibular branch, great auricular nerve, and submandibular gland, it is vulnerable to iatrogenic injury during neck dissection and rhytidectomy, which can lead to functional and aesthetic complications. This systematic review evaluates the CBFN's course, branching patterns, and surgical relevance. A PRISMA-compliant systematic review was conducted across PubMed, Web of Science, SCOPUS, and ScienceDirect. Eligible studies included anatomical or cadaveric investigations detailing the CBFN's trajectory, branching, and anastomoses. Of 402 screened records, 10 met inclusion criteria. Data were extracted on topography and inter-nerve communications. Risk of bias was assessed using a qualitative appraisal tool due to the anatomical nature of the studies. The CBFN presented as a single trunk in 61.3% to 80% of specimens and as double branches in 20% to 38.7%. Anastomoses occurred with the transverse cervical (16%-100%), great auricular (24%-38.7%), and marginal mandibular nerves (24%). The CBFN coursed deep to the platysma in all cases and inferior to the mandible, though its relation to the cervical fascia and submandibular gland varied. Injury may indirectly affect the marginal mandibular branch via anastomoses, resulting in lower lip paresis. The cervical branch exhibits substantial anatomical variation, particularly in its branching and inter-nerve connections. Intraoperative preservation requires meticulous dissection technique, and the high frequency of anastomoses highlights the need for caution during subplatysmal dissection in cervicofacial surgery. Further anatomical standardization studies are recommended to refine operative planning and improve surgical outcomes.

**Level of Evidence**: 5 (Therapeutic) 
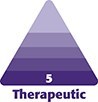

The cervical branch of the facial nerve (CBFN) innervates the platysma muscle, contributing to superficial movement of the neck and lower face contour. It typically emerges from the facial nerve trunk just inferior to the mandible, within the parotid gland, and courses inferomedially, frequently crossing over the external jugular vein before entering the superficial cervical fascia.^1^ Its proximity to structures such as the great auricular nerve and external jugular vein presents significant surgical risk during neck dissection and cervicofacial procedures.^2^

Reported anatomical studies indicate that the cervical branch usually courses deep to the platysma muscle and may divide into multiple fine terminal nerve branches supplying the platysma bilaterally.^3^ Interindividual variability in its trajectory and arborization complicates intraoperative identification. An understanding of these variations is essential to reduce the risk of iatrogenic injury. The nerve's relationship to the mandibular border and adjacent neurovascular structures underscores the need for precise dissection technique during cervicofacial surgery.^4^

The platysma plays a functional and aesthetic role by influencing neck tension and lower face dynamics. Injury to the cervical branch may result in platysmal dysfunction, lower lip droop, or asymmetry. This is particularly relevant in reconstructive and cosmetic facial procedures. Patients may present with unbalanced facial contours or an aged cervical appearance when the nerve is compromised.^5,6^ Given its critical surgical relevance, this study systematically reviews the anatomical course, branching patterns, anastomoses, and surgical implications of the cervical branch of the facial nerve, synthesizing findings from the existing anatomical literature.

## METHODS

### Study Design

This systematic review was conducted in accordance with the Preferred Reporting Items for Systematic Reviews and Meta-Analyses (PRISMA) guidelines to ensure methodological transparency and reproducibility. The objective was to synthesize anatomical literature on the cervical branch of the facial nerve (CBFN), with particular focus on its course, branching patterns, anastomoses, anatomical variability, and clinical implications relevant to cervicofacial procedures. A study protocol was drafted but not prospectively registered.

### Search Strategy

A comprehensive literature search was performed across PubMed, Web of Science, SCOPUS, and ScienceDirect. The search strategy incorporated Medical Subject Headings (MeSH) and keyword combinations including “cervical branch of facial nerve,” “facial nerve anatomy,” “surgical anatomy,” “facial nerve variations,” “neck dissection,” and “nerve preservation.” The search was limited to peer-reviewed studies published in English.

### Study Selection

Two reviewers, A.B. and L.A., independently screened titles, abstracts, and full texts against predefined inclusion and exclusion criteria. Discrepancies were resolved through discussion or consultation with a third reviewer, K.A., when necessary. Studies were eligible for inclusion if they described human cadaveric or anatomical findings pertaining to the CBFN; documented its course, variations, branching, or anatomical relationships. Exclusion criteria included studies that did not explicitly analyze the CBFN, animal experiments, reviews, editorials, case reports involving fewer than 5 specimens, conference abstracts, and clinical studies that did not provide primary anatomical data. Studies focusing solely on surgical outcomes without detailed anatomical description were also excluded.

### Data Extraction

Screening and data management were conducted using Rayyan (QCRI). For each included study, data were extracted on publication characteristics (author, year, country, study design); anatomical descriptions (nerve origin, course, branching pattern, anastomoses); and surgical relevance (landmarks and injury-prone zones).

### Risk of Bias Assessment

Risk of bias was assessed independently by the same 2 reviewers A.B. and L.A., using a qualitative appraisal approach appropriate for cadaveric anatomical studies. Each study was evaluated for clarity of dissection methodology, adequacy of sample size, and consistency of anatomical description. Disagreements were resolved through consultation with a third reviewer K.A. Other risk-of-bias tools, including the Newcastle–Ottawa Scale (NOS), were not deemed appropriate due to the descriptive, anatomical nature of the included studies.

### Data Synthesis

Findings were organized into structured evidence tables based on anatomical variation classification and the reliability of surgical landmarks. Substantial heterogeneity in anatomical classification systems, nerve identification criteria, and reporting formats precluded a valid statistical meta-analysis. Quantitative heterogeneity, assessed using the I^2^ statistic for branching patterns, was high (I^2^ > 80%), supporting a narrative synthesis approach. Subgroup tendencies were assessed by study characteristics. A qualitative synthesis was provided to highlight dominant anatomical patterns.

## RESULTS

### Study Selection

**Figure 1.**
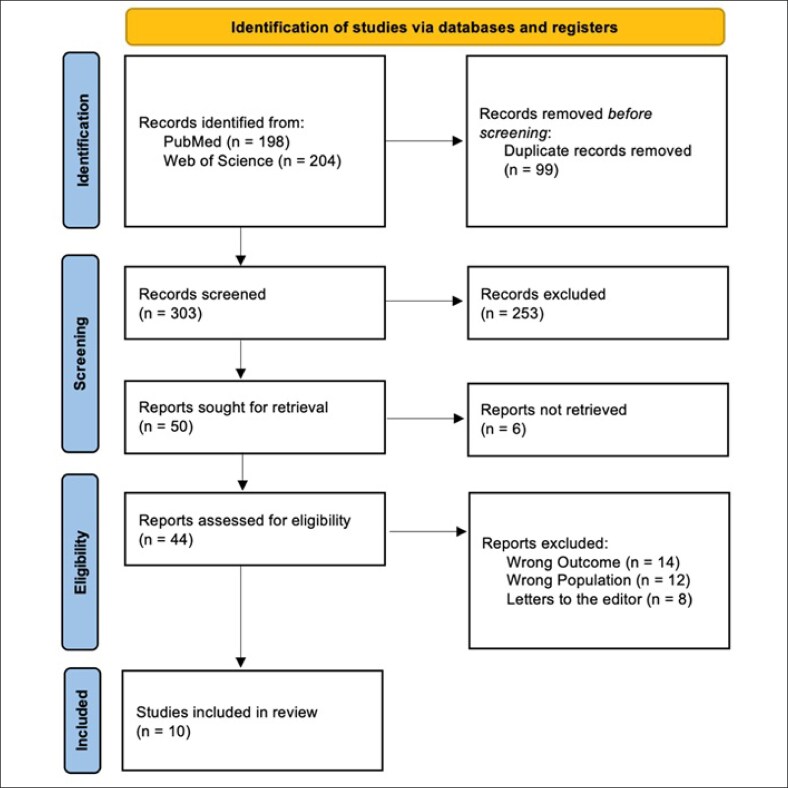
PRISMA flowchart presentation.

A total of 402 publications were initially identified through database searches. After removing 99 duplicates, 303 titles and abstracts were screened. Of these, 253 studies did not meet inclusion criteria based on anatomical relevance or study design. Fifty full-text articles were reviewed in detail. Ten studies fulfilled all eligibility criteria and were included for evidence synthesis.^7,18^ The PRISMA study selection process is shown in Figure 1, which includes a box listing the main reasons for full-text exclusion.

### Anatomical Studies of the Cervical Branch of the Facial Nerve

Of the 10 studies that met inclusion criteria, all evaluated the branching pattern of the cervical branch of the facial nerve (*n* = 10), most reported on anastomotic behavior (*n* = 8), and several described its topographic relationships to surrounding surgical landmarks (*n* = 7). These findings are summarized below.

### Branching Patterns

Across cadaveric studies, the cervical branch of the facial nerve demonstrated considerable variability in its branching configuration. Most investigations reported that the CBFN typically emerged as a single primary branch, observed in approximately 60% to 80% of specimens, as shown in the large series by Ziarah and Atkinson^7^ and supported by Righini et al.^16^ Two or more branches were present in the remaining cases. Larger series, such as Babuci et al,^18^ documented up to 5 branches, although higher-order divisions were uncommon and usually represented fine terminal arborizations supplying the platysma. Double branches were reported in 20% to 40% of dissections across multiple studies, including Cakmak et al.^11^ Triple or higher branching patterns were rare. No consistent laterality differences or sex-specific variations were identified across the included studies. Overall, the evidence indicates that while a single dominant branch is most common, surgeons may encounter a spectrum ranging from one to multiple branches during cervicofacial dissection.The variability in branching patterns and associated anastomoses across studies is summarized in Table 1.

**Table 1.** Summary of Branching Patterns and Anastomoses of the Cervical Branch of the Facial Nerve (CBFN) in Included Cadaveric Studies

Study ID	Country	No. of cadavers	No. of halves	Branching pattern	Anastomoses with
Ziarah and Atkinson, 1981^7^	UK	55	110	Single (80%), double (20%)	Transverse cervical nerve, great auricular nerve (rare)
Domet et al, 2005^8^	USA	11	22	Not reported	Transverse cervical nerve (33 anastomoses in 22 sides)
Brennan et al, 2019^9^	UK	36	72	Not reported	Not reported
Lindsey et al, 2023^10^	USA	NR	5	Not reported	Marginal mandibular branch (5/5 sides)
Cakmak et al, 2024^11^	Turkey	10	20	Single (2 sides), double (18 sides)	Transverse cervical nerve (16 sides)
Chowdhry et al, 2010^12^	USA	16	16	Not reported	Not reported
Seidel et al, 2021^13^	Germany	8	16	Not reported	Transverse cervical nerve (16 anastomoses in 16 sides)
Righini et al, 2013^14^	France	NR	NR	Single (80%), double (20%)	Transverse cervical nerve, great auricular nerve
Malins et al, 2021^15^	UK	6	11	Not reported	Not reported
Babuci et al, 2024^16^	Moldova	75	75	1-5 branches (single: 61.3%)	Transverse cervical (100%), great auricular (38.7%), marginal mandibular (24%)

NR, not reported.

### Anastomotic Patterns

Anastomotic behavior of the CBFN also showed marked heterogeneity across cadaveric studies. Communications with the transverse cervical nerve were the most frequently reported, with several investigations, particularly Domet et al^8^ and Cakmak et al,^11^ documenting multiple converging anastomoses onto a single CBFN, resulting in total anastomosis counts that exceeded the number of hemifaces examined. Additional connections were variably observed with the great auricular nerve, as noted by Ziarah and Atkinson,^7^ Righini et al,^16^ and Babuci et al,^18^ and with the marginal mandibular branch, described by Lindsey et al^10^ and reported in 24% of cases by Babuci et al.^18^ In contrast, some series, including Brennan et al^9^ and Chowdhry et al,^12^ did not identify any anastomoses. The collective findings highlight a highly variable and clinically relevant network of neural communications, underscoring the importance of meticulous dissection to avoid injury to communicating branches.

### Topographic Course

#### Relationship to the Platysma

Across cadaveric studies, the CBFN most commonly coursed deep to the platysma muscle before giving off fine terminal branches that penetrated its deep surface. Several studies, including those by Ziarah and Atkinson^7^ and Cakmak et al,^11^ noted that the main trunk descended inferiorly and medially beneath the platysma in the majority of specimens. More superficial trajectories were uncommon and typically limited to small terminal fibers rather than the primary branch. No consistent laterality differences were observed.

#### Fascial Relationships

The CBFN demonstrated variable fascial positioning along its course. In most dissections, the nerve traveled within or just beneath the superficial layer of the deep cervical fascia, particularly as it crossed the mandible and entered the upper neck. Righini et al^16^ described instances in which the nerve coursed between fascial leaflets or became adherent to the superficial cervical fascia, which may complicate surgical elevation of skin flaps. In contrast, Brennan et al^9^ and Chowdhry et al^12^ reported the nerve remaining consistently deep to the investing fascia in their samples. Collectively, the findings highlight a spectrum ranging from subplatysmal but extrafascial courses to deeper, intrafascial pathways.

#### Relationship to the Submandibular Gland

The trajectory of the CBFN relative to the submandibular gland showed notable variation across studies. Most investigations described the nerve crossing superior or lateral to the submandibular gland (SMG), frequently passing along its superior margin before arborizing in the upper neck. Domet et al^8^ and Lindsey et al^10^ reported the nerve running immediately superficial to the gland or its capsule in a subset of specimens. In contrast, Babuci et al^18^ observed cases in which the nerve coursed medial to the SMG, a configuration that may increase vulnerability during gland mobilization. Overall, although the nerve most often lies superior or lateral to the SMG, surgeons should be aware of potential positional variability.The detailed topographic relationships of the CBFN, including its course relative to the platysma, mandible, submandibular gland, and cervical fascia, are summarized in Table 2.

**Table 2.** Topographic Relationships of the Cervical Branch of the Facial Nerve (CBFN) in Included Cadaveric Studies

Study ID	Deep to platysma	Course relative to mandible	Relationship to submandibular gland (SMG)	Relationship to cervical fascia
Ziarah and Atkinson, 1981^7^	110/110	Inferior (110/110)	Not reported	Deep to fascia (reported)
Domet et al, 2005^8^	Not reported	Inferior (22/22)	Near inferior border (20/22 sides)	Not reported
Brennan et al, 2019^9^	72/72	Not reported	Not reported	Not reported
Lindsey et al, 2023^10^	5/5	Inferior (5/5)	Not reported	Superficial to fascia (5/5)
Cakmak et al, 2024^11^	20/20	Inferior (20/20)	Not reported	Deep to fascia (20/20)
Chowdhry et al, 2010^12^	16/16	Inferior (16/16)	Not reported	Not reported
Seidel et al, 2021^13^	16/16	Inferior (16/16)	Adjacent to gland (16/16)	Not reported
Righini et al, 2013^14^	Reported	Reported	Near border of gland	Deep to fascia (reported)
Malins et al, 2021^15^	11/11	Inferior (11/11)	Not reported	Not reported
Babuci et al, 2024^16^	75/75	Inferior (75/75)	Not reported	Not reported

## DISCUSSION

### Anatomical Variability

Despite sustained investigative effort, the literature presents inconsistent findings regarding branching patterns, anastomotic architecture, fascial position, and orientation relative to the submandibular gland. The subplatysmal trajectory of the CBFN is a consistent finding, confirmed by multiple studies.^7-9,18^ However, the exact fascial relationship remains debated. Cakmak et al^11^ described the nerve running deep to the investing layer of the deep cervical fascia after leaving the parotid gland, piercing the fascia distally to course within the areolar tissue beneath the platysma, ultimately terminating within the platysma muscle, whereas Lindsey et al^10^ identified it superior to this fascial layer.

Notable variability exists in the reported anatomy of the CBFN, reflecting differences in specimen characteristics, dissection techniques, and the subtle nature of the nerve's branching architecture across studies. Collectively, the literature^7-12,16-18^ demonstrates that while the CBFN follows a broadly predictable trajectory, it exhibits considerable diversity in branch number, inter-nerve communications, and relationships to regional structures. From a surgical standpoint, this variability reinforces the need for cautious dissection along the mandibular border and upper neck, with awareness that small accessory branches or unexpected anastomoses may be encountered during surgery. Rather than emphasizing isolated findings from individual studies, the overarching pattern across the evidence base highlights the importance of anticipating anatomical diversity and tailoring dissection planes accordingly during facial and cervicofacial procedures.

Topographically, Cakmak et al^11^ reported that the CBFN was located on average 6.5 mm inferior to the gonion, 7.5 mm from the anterior border of the sternocleidomastoid muscle, and approximately 18 to 21 mm inferior to the mandible, with a mean distance of 35 mm from the midline.

The nerve's orientation relative to the submandibular gland also differs among studies, with some describing a path along the gland's inferior border^8^ and others observing a more anterior trajectory.^21^ Additionally, it has been suggested that neck extension during surgery can alter this spatial relationship, increasing the difficulty of safe dissection.^22^ These differences may reflect procedural inconsistencies, interindividual variation, or underreporting. They underscore the need for meticulous intraoperative awareness and patient-specific anatomical assessment, as such variability directly influences nerve identification, dissection plane selection, and preservation strategies.

### Clinical Implications of Transverse Cervical Anastomoses

The frequent anastomosis between the CBFN and the transverse cervical nerve, as demonstrated in several anatomical studies,^7,8,11,16,18^ carries important clinical implications. These communications may provide partial motor redundancy to the platysma, which could help preserve lower facial and cervical animation in cases of proximal CBFN injury. However, these same connections increase the risk of unintended neural disruption during subplatysmal dissection, neck lift procedures, and elevation of cervical or submandibular flaps, particularly in the regions where these crossings are most often located. Awareness of these anastomotic pathways is therefore essential, as their presence may explain retained platysmal function after apparent nerve injury or, conversely, postoperative platysmal weakness despite preservation of the proximal CBFN.

### Functional Implications

The frequent interconnectivity, particularly with the marginal mandibular branch, underscores the risk of indirect lower-lip dysfunction when these connections are disrupted, even if the marginal mandibular nerve itself is preserved.^10,19,20^

Cakmak et al^11^ demonstrated that the superior or ascending branches of the CBFN, located approximately 2 cm below the mandibular angle, provide motor innervation to the depressor labii inferioris and the upper portion of the platysma complex, which act as a single functional unit responsible for lower-lip depression. Injury to these superior branches leads to weakness of the depressor labii inferioris, resulting in the loss of a full denture smile while lip eversion remains preserved due to intact mentalis function. This presentation, known as pseudo–marginal mandibular paralysis, is typically transient and tends to resolve within a few months. In contrast, injury to the marginal mandibular nerve produces persistent loss of lower-lip depression and often involves the mentalis muscle, causing chronic asymmetry that is noticeable even during gentle smiling.

### Safe Dissection Strategy

In facelift and neck lift procedures, the subplatysmal plane is generally considered a protective layer for preserving the CBFN; however, the reviewed anatomical studies demonstrate that the nerve's position can vary, with some specimens showing the CBFN superficial to or within the investing fascia.^11^ For this reason, no single plane should be regarded as universally safe. In the lateral neck, dissection is typically performed deep to the platysma and superficial to the investing fascia at an approximate depth of 4 to 5 cm below the mandibular angle, but surgeons must remain vigilant for anatomical variability. As the dissection proceeds medially, gentle blunt vertical spreading is recommended rather than traction-based or high-energy instruments. This method facilitates controlled release of cervical retaining ligaments while minimizing the risk of inadvertent injury to the CBFN, acknowledging that nerve preservation depends on respecting individual anatomical differences.

### Synthetic Anatomical Summary

**Figure 2.**
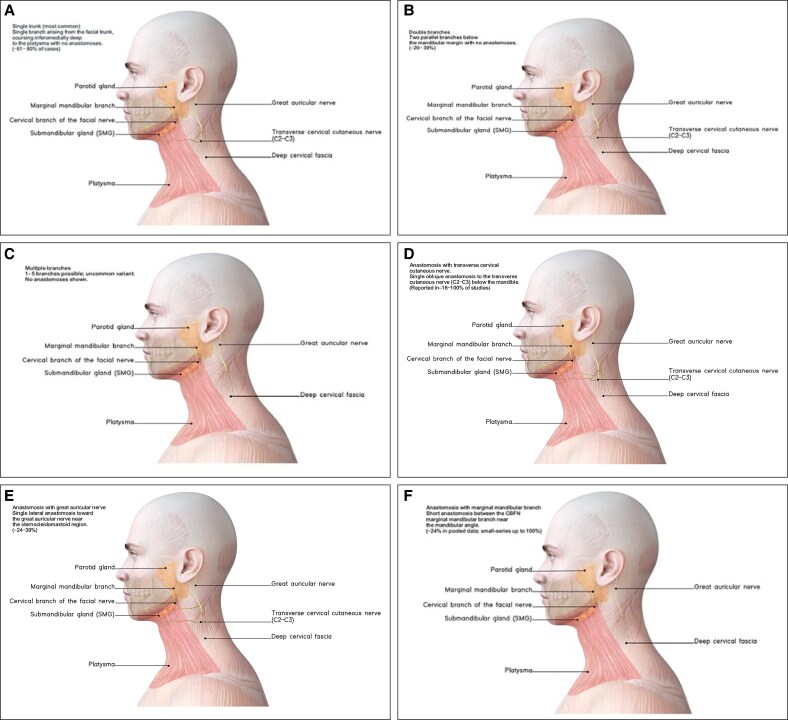
Composite anatomical variants of the cervical branch of the facial nerve (CBFN) based on pooled cadaveric findings. 2025 Simona Molino. Reproduced with permission. (A) Single-branch configuration: a solitary primary trunk coursing deep to the platysma before arborizing into terminal fibers. (B) Double-branch configuration: 2 primary branches emerging from the facial nerve and descending toward the upper neck. (C) Multiple-branch configuration: three or more primary branches with fine terminal arborizations supplying the platysma. (D) Anastomosis between the CBFN and the transverse cervical nerve. (E) Anastomosis between the CBFN and the great auricular nerve. (F) Anastomosis between the CBFN and the marginal mandibular branch.

Based on the aggregated findings, a synthetic anatomical summary is proposed (Figure 2). The most frequent configuration (approximately 60%-80% of cases) involves a single CBFN trunk originating from the main facial nerve trunk, coursing inferomedially deep to the platysma muscle, and running inferior to the mandibular border. It commonly exhibits anastomoses with the transverse cervical nerve. A significant variant (approximately 20%-40%) features a double-branch pattern. Less frequent variations include anastomoses with the great auricular nerve or, more rarely, the marginal mandibular branch. The relationship to the submandibular gland is variable, often reported near its inferior pole, and the nerve's position relative to the deep cervical fascia can be superficial, deep, or coursing within the superficial layer of the fascia, depending on the specimen.

### Future Directions

Based on expert opinion, high-resolution ultrasound may enhance surgical precision by enabling real-time visualization of peripheral nerve branches, fascial relationships, and anastomotic pathways.^23^ Such adjunctive imaging could reduce the risk of inadvertent injury and serve as a dynamic complement to static cadaveric studies, although its role in mapping the CBFN has not been systematically evaluated. Future research should prioritize standardized cadaveric protocols with consistent definitions of fascial planes, branching classifications, and anastomotic terminology. Integration of imaging validation and clinical correlation will further clarify the anatomy of the CBFN and support refinement of nerve-preservation strategies.

### Limitations

This review has several important limitations that must be acknowledged. First, the total number of eligible studies was small, limiting the robustness and generalizability of the synthesized findings. Considerable heterogeneity existed among the included studies in terms of dissection technique, measurement methodology, depth of anatomical detail, and reporting standards, which restricted the ability to perform quantitative pooling. Nearly one-third of all hemifaces analyzed arose from a single large study,^18^ introducing disproportionate weighting of its anatomical patterns relative to other sources. Several studies also provided incomplete data; for example, Righini et al^16^ did not report the number of specimens examined, and others omitted key metrics such as nerve depth, fascial relationships, or the precise nature of anastomotic connections. Additionally, cadaveric studies inherently vary in tissue condition, preservation technique, and demographic representation, all of which may influence the observed anatomy. These factors collectively limit the external validity of the conclusions and underscore the need for standardized, high-resolution anatomical investigations.

## CONCLUSIONS

The cervical branch of the facial nerve displays substantial anatomical variability in its branching, anastomotic behavior, and spatial relationships to surrounding structures such as the platysma, submandibular gland, and cervical fascia. Its consistent course deep to the platysma and frequent interconnections with nerves including the transverse cervical and marginal mandibular branches place it at elevated risk during cervicofacial surgery. Future studies employing standardized cadaveric protocols and consistent anatomical definitions are essential to refine understanding of this nerve's morphology and to support improved operative planning and outcomes.
